# The soil depth determines soil multifunctionality *via* shaping the soil properties and microbial diversity

**DOI:** 10.7717/peerj.20734

**Published:** 2026-02-16

**Authors:** Xiaoxia Liang, Yunxiao Zhao, Yanhua Song, Baofeng Chai, Tong Jia

**Affiliations:** Shanxi University, Taiyuan, China

**Keywords:** Soil depths, Soil properties, Microbial diversity, Microbial network complexity, Multifunctionality

## Abstract

**Background:**

Soil microorganisms drive subsurface ecological processes and are shaped by soil properties, which in turn influence biogeochemical cycling. Although the link between biodiversity and soil multifunctionality (SMF) has received widespread attention, the relative roles of soil properties and microbial community structure in regulating SMF remain unclear.

**Methods:**

Here, we investigated SMF and microbial communities in the humus layer and across the 0–80 cm depth of natural soil profiles in a subalpine grassland using high-throughput sequencing.

**Results:**

Our findings revealed that SMF, microbial diversity and network complexity decreased significantly with soil depth. Microbial community structure was primarily determined by pH and soil water content (SWC). Soil properties were the primary drivers of SMF, predicting 47.24%–63.75% of its variance. Microbial diversity was a stronger predictor of SMF than network complexity, explaining 26.09–44.56% of its variation. Bacterial diversity was significantly positively correlated with soil nutrient, carbon and nitrogen multifunctionality, while fungal diversity was not significantly correlated with them. This finding provides critical data support for elucidating the relationship between biodiversity and ecosystem functioning.

## Introduction

Since its proposal in the 1990s, the biodiversity-ecosystem functioning (BEF) theory has highlighted the crucial role of biodiversity in sustaining ecosystem productivity and stability. Research has increasingly shifted from examining single ecosystem functions to a multifunctional perspective, emphasizing that biodiversity supports multiple ecosystem services simultaneously (Hector & Bagchi, 2007; Manning et al., 2018; Yang et al., 2019). Within this framework, soil multifunctionality (SMF) serves as a comprehensive index that integrates multiple soil functions and reflects various belowground processes (Wagg et al., 2014). As core drivers of subsurface ecosystem processes, soil microorganisms are central to understanding SMF (Luo et al., 2023a). Their diversity and interactions (*e.g.*, predation, competition) are thought to significantly influence SMF (Hu et al., 2024; Mao et al., 2024; Wang et al., 2023). However, the relationship between soil microbial diversity, interactions and SMF remains controversial (Delgado-Baquerizo et al., 2017; Zhou, Wang & Luo, 2020).

Some researches have shown that higher microbial diversity promotes the decomposition of organic matter through functional redundancy, metabolic complementarity, thereby maintaining the stability of soil multifunctionality (Huang et al., 2021). Previous studies argue that microbial diversity and SMF are negatively (Zhou, Wang & Luo, 2020), positively (Canarini et al., 2021), or nonsignificantly linked (Zhou et al., 2024). Interactions among soil microorganisms may influence ecosystem functions due to population fluctuations, and the complex ecological interactions among them significantly affect the resistance and resilience of ecosystems to environmental changes (Dina et al., 2023; Ramirez et al., 2018; Weiai et al., 2021). The loss of microbial network complexity may be detrimental to ecosystem health and affect SMF (Luo et al., 2023b). Many studies have further revealed that microbial network complexity (*e.g.*, modular structure, key species connectivity) can reflect community stability (Jiao et al., 2022a; Maynard, Crowther & Bradford, 2017). For example, a highly connected bacterial network can enhance the drought resistance of soil ecosystems, while the modular features of fungal networks may alleviate the negative impact of nutrient competition on SMF (Long et al., 2025). Moreover, the relationship between diversity and interaction networks and ecosystem functions may differ between bacterial and fungal communities due to differences in their biological characteristics, ecological niches, and life history strategies (Bahram et al., 2018; Gaüzère et al., 2022; Song et al., 2014; Zhang et al., 2024). Higher bacterial community diversity may enhance functional diversity, thereby supporting multiple ecosystem functions (Chen et al., 2023). In contrast, fungi—through unique reproductive strategies such as spore production and extensive hyphal networks—are thought to strengthen species interactions, making their interaction networks a key driver of ecosystem functioning (Bonfante & Genre, 2010; Martin & Van der Heijden, 2024).

Notably, microbial community structure is not the only factor affecting SMF. Abiotic factors, particularly soil pH and water content, also play a key role in determining SMF. Soil pH influences SMF by regulating organic matter dynamics, nutrient availability, and microbial diversity (Chen et al., 2018; Li et al., 2021; Delgado-Baquerizo et al., 2020). Soil water content affects plant–soil interactions and biogeochemical cycling, thereby modulating SMF (Li et al., 2022). Although depth-related patterns of soil properties, microbial communities, and SMF have been reported in various ecosystem (Li et al., 2024; Yang et al., 2023), studies focusing on the humus layer and deep soils (>40 cm) in subalpine grassland remain scarce. Subalpine grasslands are increasingly degraded by climate change and intense human activities, leading to declining soil productivity (Michalet et al., 2024). Therefore, exploring the relative importance of microbial community structure and soil properties in subalpine grasslands to assess SMF is crucial for predicting ecological consequences and rationally formulating conservation measures.

In this study, we investigated microbial community and SMF from the vertical soil profile, *e.g.*, humus layer, 0–10, 10–20, 20–40 and 40–80 cm cross-layer in the subalpine grassland in Luya Mountain, Shanxi Province, China. We aim to explore the driving factors of SMF and microbial community in different depth, and the relationship between the soil bacterial and fungal communities diversity, network complexity, soil properties and SMF. Thus, two hypotheses have been proposed: (1) SMF and microbial community diversity decreased along the soil depth, influenced by physicochemical properties; (2) microbial diversity were more strongly associated with SMF than network complexity. The results elucidate the drivers of soil multifunctionality and provide a theoretical basis for predicting ecosystem service trajectories in subalpine grassland.

## Materials & Methods

### Study site

This work was carried out at Subalpine Grassland Ecosystem Observation and Research Station of the Ministry of Education (Shanxi, China), which is situated in Luya Mountain (E 111°50′22′, N 38°43′43′) in the Lvliang Mountains of Shanxi Province. The altitude is 2,754 m, the annual average temperature is 6–10 °C, the annual average precipitation is 380–680 mm (Zhang et al., 2018), the annual average relative humidity is 50%–55%, and the annual evaporation is 1,800 mm (Bai et al., 2018). The location experiences a temperate continental monsoon climate (Xue et al., 2020). The main vegetation type is subalpine grassland, dominated by *Carex*, with associated species including *Gentiana* and *Polygonum viviparum* L.

### Soil sample collection

In early August 2021, we established three spatially independent sampling plots in a subalpine grassland on Luya Mountain, China, with a minimum distance of 50 m between plots to minimize spatial autocorrelation. At each plot, an approximately 80-cm-deep soil profile was excavated, and samples were collected from five depth layers: the humus layer (C0), 0–10 cm (C1), 10–20 cm (C2), 20–40 cm (C3), and 40–80 cm (C4). Within each layer, ten soil cores were collected using a sterile auger and homogenized into a single composite sample per layer per plot. Samples were immediately placed in sterile plastic bags, stored on ice during transport, and delivered to the laboratory within 24 h. All sampling tools were sterilized with 75% ethanol to prevent cross-contamination. Visible plant roots and stones were removed by hand, and the soils were then sieved through a 2-mm mesh. Subsamples were allocated as follows: (i) air-dried for soil physicochemical analyses; (ii) stored at 4 °C for biological assays; and (iii) frozen at –20 °C for microbial community analysis *via* high-throughput sequencing.

### Soil physicochemical properties and enzyme activity determination

Soil pH was measured using a pH meter (HAANNA H13221) (point method, 1:2.5 soil-water ratio); fresh soil samples were loaded into aluminum boxes and dried to a constant weight in an oven at 80 °C to determine soil water content (SWC); soil total nitrogen content (TN), soil total carbon content (TC) and soil total sulfur content (TS) were determined by elemental analyzer (varioMACRO cube, varioMACRO, Langenselbold, Germany). Soil total phosphorus content (TP) was assayed using the sulfuric acid-perchloric acid digestion method followed by the molybdenum-antimony anti-colorimetric method, utilizing an automatic analyzer. Soil nitrate nitrogen (NO_3_^−^-N) and ammonium nitrogen (NH_4_^+^-N) were determined with an automated discrete analyzer (CleverChem 380; CleverChem, Hamburg, Germany) using the hydrazine reduction method for NO_3_^−^–N and the KCl extraction–indophenol blue colorimetric method for NH_4_^+^–N, respectively.

Enzyme activities were measured using standardized protocols: N-acetyl-β-D-glucosidase (S-NAG), β-glucosidase (β-GC), and leucine aminopeptidase (L-LAP) by microplate fluorescence; polyphenol oxidase (PPO) and peroxidase (POD) by the L-DOPA method; and neutral phosphatase (NP) by disodium phenyl phosphate colorimetry. Incubations were performed at 37 °C for 1 h (30 °C for PPO and POD). Activities are expressed as units per gram of dry soil (U g^−^^1^), where 1 *U* = 1 µmol substrate hydrolyzed per minute. All assays were conducted in triplicate by Bioengineering Co., Ltd., and mean values were used for statistical analysis, following established methods (Saiya-Cork, Sinsabaugh & Zak, 2002).

### DNA extraction, high-throughput sequencing and bioinformatics analysis

A quantity of 0.25 g of homogenized soil samples were weighed for DNA extraction using the E.Z.N.A.^®^ soil DNA kit (Omega Bio-tek, Norcross, GA, USA). The 16SrRNA gene in bacteria was amplified by primers 338F (5′-ACTCCTACGGGAGGCAGCAG-3′) and 806R (5′-GGACTACHVGGGTWTCTAAT-3′) (Xu et al., 2016), and the ITS gene in fungi was amplified using primers ITS1F (5′-CTTGGTCATTTAGAGGAAGTAA-3′) and ITS2R (5′-GCTGCGTTCTTCATCGATGC-3′) (Adams et al., 2013). The primers TAReuk454FWD1F (5′-CCAGCASCYGCGGTAATTCC-3′) and TAReukREV3R (5′-ACTTTCGTTCTTGATYRA-3′) (Stoeck et al., 2010) were used to amplify the 18S rRNA protists gene. We submitted the raw sequencing data to the National Center for Biotechnology Information (NCBI) Sequence Read Archive (SRA) (https://www.ncbi.nlm.nih.gov/sra, accessed on 7 February 2023) under project accession number PRJNA932581. For PCR amplification details see Supplemental Files S1.

### Microbial co-occurrence network construction and analyses

Co-occurrence network analysis was used to reflect the coexistence of microbial communities at varying soil depths. In order to avoid the influence of insufficient sample size on the co-occurrence network, the data of 0–10 cm layer (C1) and 10–20 cm layer (C2) were merged to construct the surface soil microbial molecular ecological network, and the data of 20–40 cm layer (C3) and 40–80 cm layer (C4) were merged to construct the sub-top soil microbial molecular ecological network. At the same time, five soil depth gradients (C0 + C1 + C2 + C3 + C4) data were combined to construct a subalpine grassland soil microbial molecular ecological network to explore its complexity and key taxa.

Operational taxonomic units (OTUs) with Spearman correlation coefficients >0.6 and *p* < 0.05 were retained in the construction of the soil microbial co-occurrence network (Liu et al., 2021). This data screening approach ensures that OTUs with possible interactions can be identified, which minimizes potential for spurious correlations. The topological attributes of the co-occurrence network were then calculated and visualised using Gephi 0.9.3. This study identifies the OTUs corresponding to the top five betweeness centrality as key taxa. Furthermore, this study utilized edge density, which correlates strongly with the number of nodes and edges that characterise the complexity of microbial networks, as a measure of network complexity in microorganisms (Yuan et al., 2021). All statistical analyses were performed in R (v4.2.0.1; R Core Team, 2022). Other details see Supplemental Files S1.

### Assessing soil multifunctionality

Since different types of elements have distinct function patterns that may be counteracted by combining the elements, we calculated the multifunctionality of nutrient, carbon and nitrogen separately. Specifically, soil nutrient multifunctionality was evaluated using TN, TC, TS, TP, and their stoichiometric ratios (C:N, C:P, N:P). Soil carbon multifunctionality was assessed based on TC, C:N ratio, and the activities of extracellular enzymes involved in carbon decomposition—β-GC, PPO and POD—which reflect key processes in the degradation of cellulose and lignin (Deng & Tabatabai, 1995; Feyissa et al., 2022; Jian et al., 2016). Soil nitrogen multifunctionality was quantified using TN, C:N and N:P ratios, NO_3_^−^-N, NH_4_^+^-N, as well as the enzymes S-NAG and L-LAP, which are critical for the decomposition of organic nitrogen sources such as chitin and proteins (Feyissa et al., 2025; Luo, Meng & Gu, 2017; Tabatabai & Bremner, 1972).

Soil multifunctionality was evaluated using two complementary approaches implemented in the “multifunc” R package (v0.8.0): (1) the averaging approach, which calculates multifunctionality as the mean of all max-normalized ecosystem functions; and (2) the multiple-threshold approach, which quantifies the proportion of functions exceeding a given threshold (expressed as a percentage of each function’s observed maximum). For the latter, we assessed thresholds ranging from 5% to 99% at 1% intervals to examine the sensitivity of multifunctionality (Byrnes et al., 2014) to threshold choice.

### Data analysis

One-way analysis of variance (ANOVA) analysis and Duncan’s multiple range test were employed to compare the differences in SMF, physicochemical properties and enzyme activities at different soil depths. Non-metric multidimensional scaling analysis (NMDS) was performed on bacterial and fungal communities between different soil depths using a Bray-Curtis-based distance algorithm. The stress value <0.05, indicating an excellent fit of the NMDS ordination and that it reliably represents the true dissimilarities among microbial communities. In addition, VIF (variance inflation factor) analysis was used to filter out environmental factors with strong multicollinearity, and redundancy analysis (RDA) or canonical correspondence analysis (CCA) was applied to illustrate the relationship between microorganisms, environmental factors and soil depth. The selection basis is detailed in Supplemental Files 1. Spearman correlation analysis was performed to identify the key environmental factors affecting the microbial community. Structural equation model was constructed to explore the direct and indirect effects of soil depth and environmental factors on microbial community structure. The relationship between microbial diversity, network complexity and soil multifunctionality was analyzed by general linear expression models (GLMs) based on lm function in R. Random forest model was used to evaluate the relationship between soil physicochemical properties, enzyme activity and biotic factor on soil multifunctionality based on the “randomForest” package. The mixed-effects model was used to evaluate the effects of soil properties, microbial diversity and network complexity on soil multifunctionality through the “lme4” package.

## Results

### Soil physicochemical properties and enzyme activities at different depths

**Figure 1:**
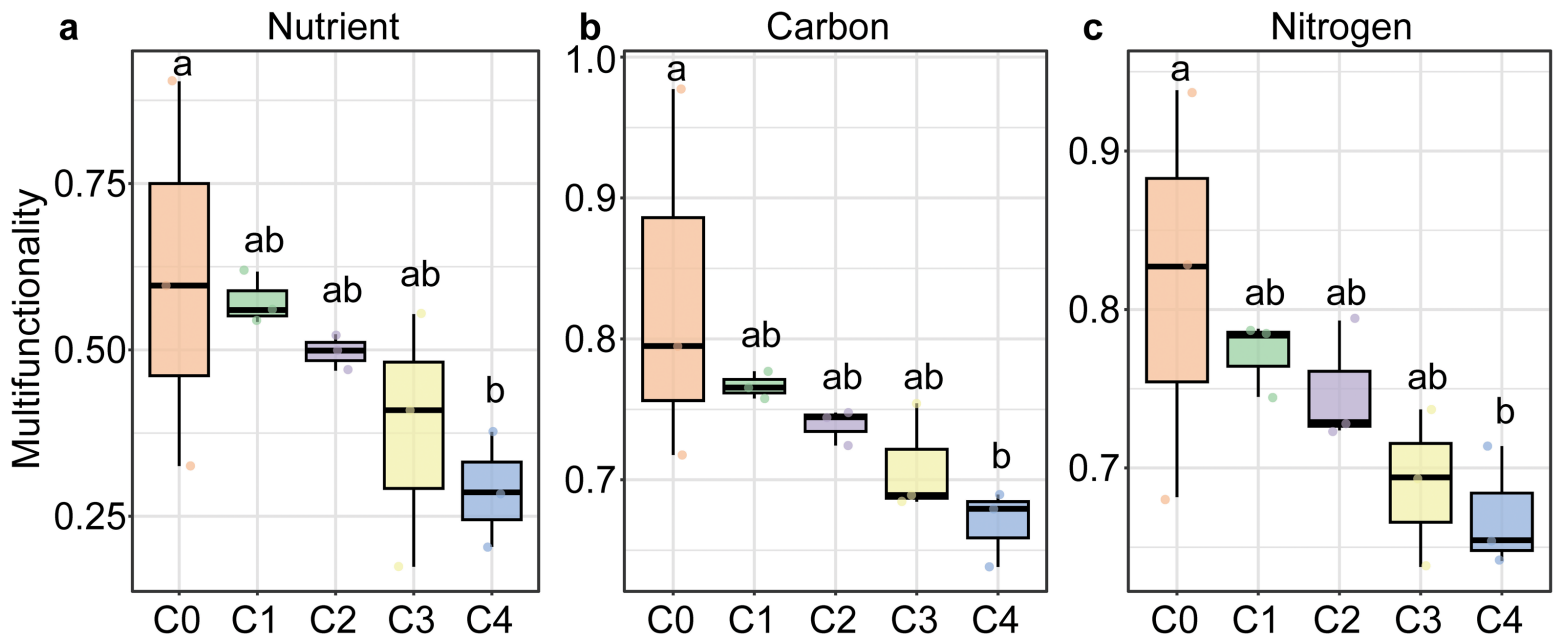
Soil multifunctionality along soil depths in the subalpine grassland in Luya Mountain. Different letters on the box plot indicated significant differences between soil depths. C0: humus layer; C1: 0–10 cm; C2: 0–10 cm; C3: 20–40 cm; C4: 40–80 cm.

All three soil multifunctionality significantly decreased with soil depth, showing the highest values in the humus layer (C0) and the lowest in the 40–80 cm layer (*P* < 0.05; Fig. 1). The soil of *Carex* subalpine grassland in Luya Mountain was weakly acidic (pH, 5.823−6.613). SWC of the topsoil (0–10 cm) was significantly higher than that of the deeper soil layers. TN and TS decreased significantly with soil depth, peaking at 5.265 g kg^−1^ and 0.787 g kg^−1^ in the 0–10 cm layer, respectively. In contrast, TC was the highest in the humus layer (71.308 g kg^−1^) and also declined significantly with depth. NH_4_^+^-N content was highest in the 20–40 cm soil layer (93.39 mg kg^−1^) and lowest in the 40–80 cm layer (37 mg kg^−1^), with significant differences among layers (*P* < 0.05; Fig. S1). However, there was no significant difference in TS, NO_3_^−^-N and enzyme activity at different depths of soil profile (*P* > 0.05, Table S1).

### Structure of soil microbial community and influencing factors at different depths

**Figure 2:**
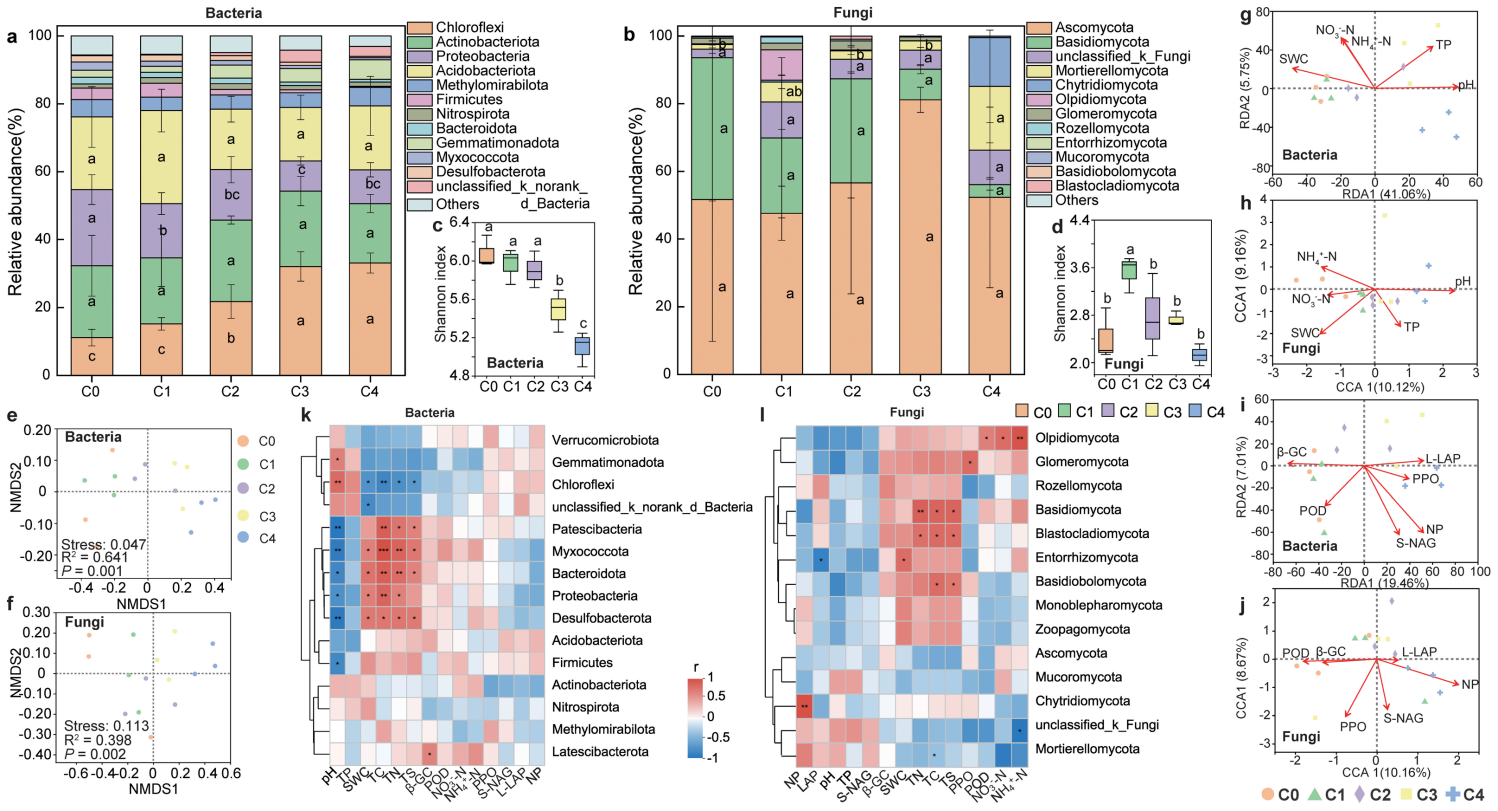
Soil microbial community composition, diversity and influencing factors at different depths. Top 12 soil bacterial (A) and fungal (B) phylum of communities, and microbial communityα-diversity (C–D) and β-diversity (E–F) at different soil depths. RDA and CCA analysis (G–J) of bacterial and fungal communities with soil physicochemical factors and enzyme activities, respectively. Spearman correlation analysis of bacterial (K) and fungal (L) communities of top15 with soil physicochemical factors and enzyme activities, respectively. (Significant differences are expressed as: * *P* < 0.05, * * *P* < 0.01, * * * *P* < 0.001). C0: humus layer; C1: 0–10 cm; C2: 0–10 cm; C3: 20–40 cm; C4: 40–80 cm.

The abundance of both bacterial and fungal community exhibited an increasing and then decreasing trend along the soil depth (Fig. S1). The dominant bacterial phyla in the five soil layers were Chloroflexi, Acidobacteria, Proteobacteria, Actinobacteriota. Among them, the relative abundance of Chloroflexi increased significantly with soil depth, whereas that of Proteobacteria decreased significantly (*P* < 0.05; Fig. 2A). Ascomycota, Basidiomycota, unclassified_k_Fungi, and Mortierellomycota were the dominant fungal phyla in soil depth, collectively accounting for 85.11% of the fungal community. And the relative abundance of Ascomycota significantly increased with soil depth (*P* < 0.05; Fig. 2B, Figs. S3, S4). Both bacterial and fungal community diversity declined gradually with soil depth. Notably, bacterial diversity significantly exceeded that of fungi across all layers (Figs. 2C, 2D). NMDS ordination revealed distinct clustering of bacterial community across soil depths. Both bacterial (*R*^2^ = 0.6408, *P* < 0.001) and fungal (*R*^2^ = 0.3975, *P* < 0.05) communities structures differed significantly in soil depth (Figs. 2E, 2F).

Microbial communities across soil layers were influenced by various soil physicochemical properties and enzyme activities. RDA analysis indicated that pH, SWC and TP were the key factors affecting the bacterial community at different depths (explained 46.81% of the total variation) (Fig. 2G). In contrast, fungal community were primarily influenced by pH, SWC, and NH_4_^+^-N (explained 19.28% of the total variation) (Fig. 2H). CCA analysis identified NP and PPO as the major enzyme activities affecting soil fungal community structure (explained 18.83% of the total variation) (Fig. 2J).

Spearman correlation analysis revealed significant associations between soil bacterial communities and SWC, pH, and TC. Chloroflexi was significantly negatively correlated with SWC, whereas Firmicutes, Proteobacteria, and Bacteroidota were positively correlated with SWC (*P* < 0.01). In addition, Chloroflexi was positively correlated with pH, while Firmicutes and Proteobacteria showed negative correlations with pH (*P* < 0.05). A significant negative correlation was also observed between Chloroflexi and TC, whereas Firmicutes and Proteobacteria were positively correlated with TC (Fig. 2K). For the fungal community, significant correlations were detected with TC, TS, TN, NH_4_^+^-N, and POD. Basidiomycota showed positive correlations with TC, TS, and TN. Chytridiomycota was positively correlated with NP, while Glomeromycota was positively correlated with PPO. Olpidiomycota was significantly positively correlated with POD, NO_3_^−^-N and NH_4_^+^-N. In contrast, Mortierellomycota was negatively correlated with NH_4_^+^-N (Fig. 2L).

### The co-occurrence relationship of soil microbial community in different soil depth

**Figure 3:**
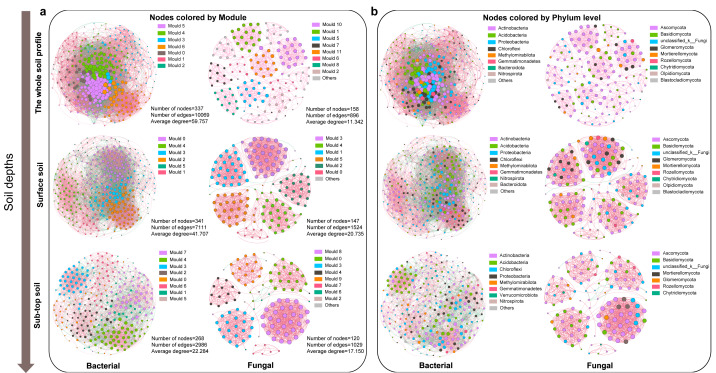
Co-occurrence network of microbial community based on module (A) and phylum level (B) in different soil layers. The node color represents the microbial classification unit to which the OTU belongs (here divided at the module and phylum level). Node size represents the degree of different module or microbial phylum. The pink edge represents positive correlation, and the green edge represents negative correlation.

The bacterial co-occurrence network was more complex than that of fungi (Fig. 3). The node, edge number and edge density of bacteria and fungi showed a decreasing trend along the soil profile (Fig. S5, Table S2). The bacterial and fungal networks in surface soil were more densely connected and structurally complex compared to those in sub-top soil. The modularity of bacterial community in surface layer was lower than that in sub-top layer, while that of fungi was the opposite. Therefore, bacterial community in sub-top soil and fungal community in surface soil demonstrated higher functional differentiation and greater stability under disturbance (Fig. 3, Table S2). In addition, the microbial co-occurrence networks of surface and sub-top soil were mainly positive correlation, and the positive correlation coefficient increased with the soil depth (Table S2). The key bacterial taxa were Thermoleophili, Acidimicrobiia and Methylomirabilia, and key fungal taxa were Sordariomycetes and Dothideomycetes. Actinobacteriota in surface soil and Ascomycota in sub-top soil play a potentially vital role in maintaining microbial community structure and function (Table S3).

The environmental factors influencing the key taxa of soil microbial network were further analyzed by Mantel test (Fig. S6). The results indicated that soil pH, SWC, S-NAG, and β-GC significantly affected the microbial community composition of key taxa. As well, soil pH, TN, TC, and TS were identified as the core ecological factors affecting the composition and diversity of soil bacterial and fungal communities (Table S4 and Fig. S6). Based on the structural equation model, TC emerged as the primary determinant of bacterial and fungal community structure and diversity, followed by soil depth, pH, and SWC (Fig. S7).

### The relative importance of microbial diversity, network complexity, soil properties on soil multifunctionality

**Figure 4:**
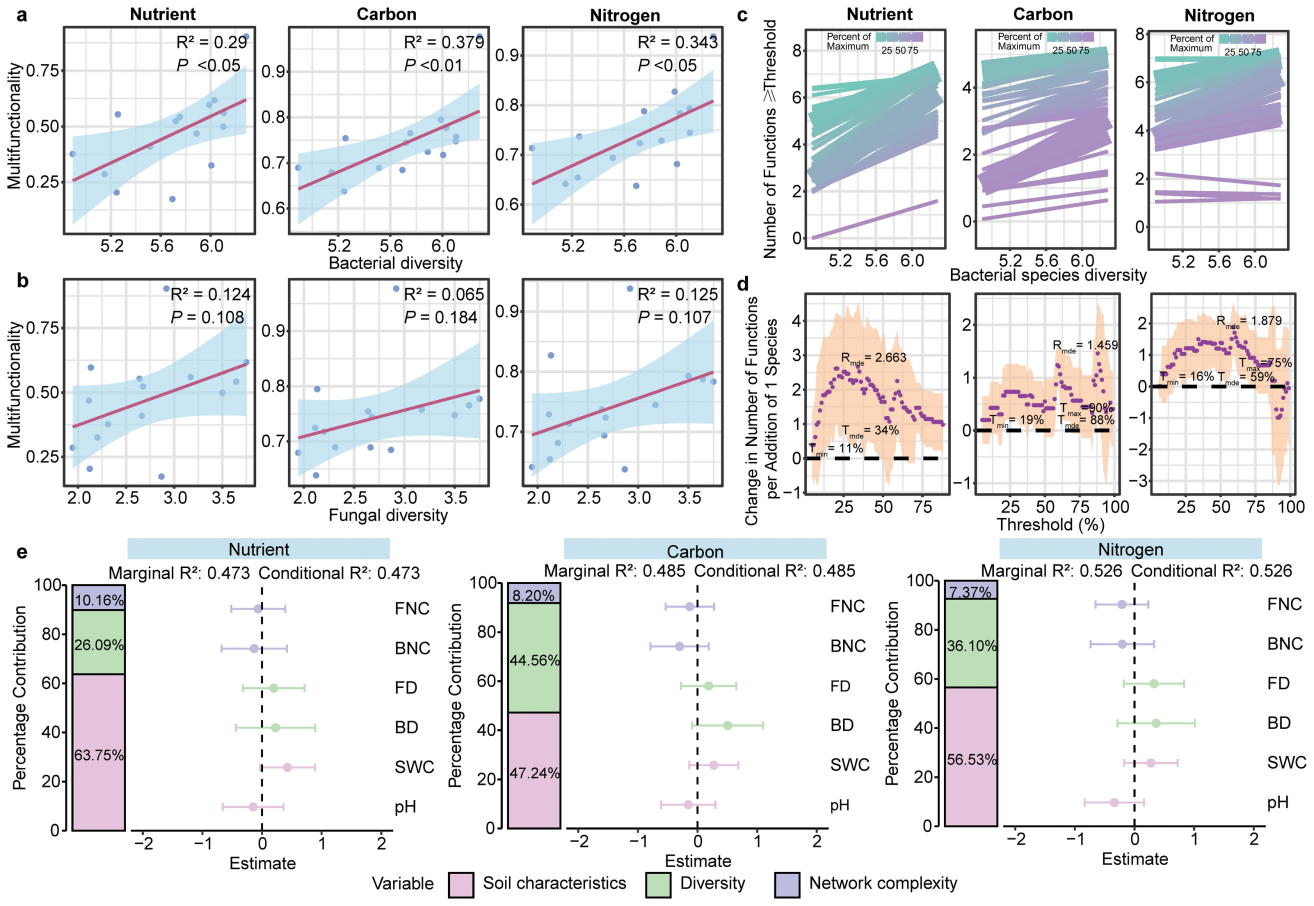
Linkage between microbial diversity, network complexity, soil properties and soil multifunctionality. The relationship between bacterial (A) and fungal (B) community diversity and multifunctionality. (C) Relationship between bacterial diversity and function with different thresholds in different ecosystem multifunctionality categories. The relationship between bacterial diversity and the number of functions is equal to or greater than the observed functions of different proportions. The solid line is the fitting relationship of the GLMs; the gradient colour represents the maximum proportion of functions. (D) The slope between bacterial diversity and different threshold functions. The purple solid point is the fitting value of the GLMs; the shaded part represents the ±1 confidence interval. Tmin (the lowest threshold where diversity-multifunctionality relationships become significant), Tmax (the highest threshold where diversity-multifunctionality relationships become significant) and Tmde (the threshold where the diversity-multifunctionality relationship is strongest). Rmde (the realised maximum effect of diversity). These “threshold” refers to the proportion (%) of each ecosystem function’s maximum observed value across all samples. We evaluated thresholds from 5% to 99% to examine how the relationship between bacterial diversity and multifunctionality varies with the stringency of functional performance criteria. (E) The relative effects of soil microbial diversity, network complexity and soil properties on SMF based on mixed-effects model.

GLMs showed a positive association between bacterial community diversity and three multifunctionality (nutrient, carbon and nitrogen) (*P* < 0.01; Fig. 4A). However, fungal diversity was not associated with any multifunctionality (*P* > 0.05; Fig. 4B, Fig. S8). Furthermore, the results of the multiple threshold analysis revealed that the minimum (Imin, the minimum functional threshold where multifunctionality begins to be affected by diversity) and maximum (Tmax, SMF beyond which multifunctionality is not affected by diversity) functional thresholds of bacteria ranged from 11% to 90%, while the threshold of maximum diversity effect (Imde) was 88% for the bacterial community. At this point, the realized maximum effect of diversity (Rmde, *i.e.,* slope) reached 1.459 (Figs. 4C, 4D). For fungal communities, however, the Rmde was only 1.005 (Fig. S9).

Random forest analysis identified soil physicochemical properties, bacterial diversity and network complexity as key predictors of the soil multifunctionality (Fig. S10). To further explore the effects of soil properties, microbial diversity, and network complexity on SMF—while avoiding multicollinearity between soil variables and multifunctionality, for which only pH and SWC were retained as soil property proxies—mixed-effects models were constructed for each of the three multifunctionality types. Soil physicochemical properties were positively correlated with multifunctionality, with pH and SWC explaining 47.24–63.75% of the variance in SMF. Microbial diversity emerged as a stronger biotic driver of multifunctionality than network complexity across three multifunctionality. In particular, microbial diversity significantly influenced carbon cycle multifunctionality, explaining 44.56% of the variance (Fig. 4E).

## Discussion

In this study, we found that soil depth had a profound impact on SMF and microbial community structure. Soil properties (pH and SWC) are the key predictors of SMF. Specifically, regulated by soil properties, microbial diversity and SMF decreased significantly along the soil depth (*P* < 0.05), which supported the first hypothesis. Consistent with the second hypothesis, microbial diversity is more strongly correlated with SMF than network complexity (*R*^2^ = 0.473−0.526, *P* < 0.05). These results indicate that soil depth determines SMF by shaping soil properties and microbial diversity.

### Soil multifunctionality and microbial diversity decline with increasing soil depth

As expected, the SMF in the surface layer of the subalpine grassland was significantly higher than that in the sub-top layer (Fig. 1). Similar results have been observed in farmland (Zhang et al., 2021) and forest (Ding et al., 2024) ecosystem. Ding et al. (2024) found that the water-holding capacity of deep soil plays an important role in affecting multifunctionality. Compared with the topsoil, the input of plant-derived debris decreased rapidly in the sub-top soil (Ma et al., 2020), and the roots (>30 cm) distributed in the subsoil accounted for less than 20% of the grassland underground biomass (Wang et al., 2020), which may lead to a decrease in soil water content, thereby affecting the release of effective soil nutrients (Xu et al., 2023). In other words, deep soil is less influenced by vegetation and litter input, but it is still limited by soil moisture, which in turn affects multifunctionality (Xu et al., 2023). Moreover, soil depth significantly influences soil nutrient content and stoichiometry (Chen et al., 2022b). The organic matter content and nutrient utilization rate of topsoil are higher than those of subsoil, which may contribute to enhanced SMF (Yao et al., 2025). In addition, changes in microbial community structure and diversity also indirectly affect SMF driven by certain soil nutrient changes (Cui et al., 2022; Zhou, Wang & Luo, 2020). Our results showed that soil microbial diversity decreased with the increase of soil depth, which also confirmed the above statement. However, soil microbial community structure is not the only factor affecting multifunctionality (Giling et al., 2019).

### Soil properties are the key drivers of soil multifunctionality

Previous studies have suggested that soil environmental variables (such as pH, SWC and texture) are good predictors of soil carbon and nitrogen cycling processes and play a key role in determining SMF (Graham et al., 2016). Our results support this argument that soil properties (pH and SWC) are key drivers of soil multifunctionality, with variance explained rates as high as 47.24%–63.75% (Fig. 4). Soil pH increased with depth, and was significantly negatively correlated with SMF (Fig. S10). This can be attributed to two main factors: first, the ammonia oxidation activity of the surface soil was higher than that of the sub-top soil, resulting in the production of a vast array of hydrogen ions through the oxidation of ammonia to nitrate (Guo et al., 2023). Second, it was linked to the accumulation of phenolic acids present in a considerable amount of root exudates in the surface soil (Ullah et al., 2023; Yang et al., 2018). This study also found that soil water content and nutrient content decreased significantly with the increase of soil depth, and were significantly positively correlated with SMF (Fig. S10). Similar results were found in grassland (Will et al., 2010), paddy field (Li et al., 2017), drylands (Semenov et al., 2018) and forest ecosystems (Du et al., 2025). This is due to the ability of soil moisture to promote multiple ecosystem functions such as material degradation, metabolic cycle (Li et al., 2022) and soil nutrient accumulation (Yang et al., 2023), especially in arid and semi-arid grasslands dominated by precipitation. Previous studies have shown that soil moisture plays a crucial role in shaping the ecosystem structure and function of alpine swamp meadows dominated by groundwater (Yang et al., 2023; Zhao et al., 2019). The decline of groundwater level will affect species distribution patterns, plant productivity and soil nutrient cycling, which will have a negative impact on the ecological function of alpine swamp meadow (Zhao et al., 2022).

At the same time, a wide range of soil variables such as soil pH, humidity and nutrient content will affect the composition and diversity of soil microbial communities, and thus indirectly affect SMF (Brockett, Prescott & Grayston, 2012; Richter et al., 2018). Globally, pH is considered to be a key predictor of soil microbial community composition and diversity (Zhalnina et al., 2015). With the aggravation of soil acidification, soil solid aluminum will be transformed into exchangeable Al^3+^, and the increased Al^3+^ will inhibit extracellular enzyme activity and produce physiological stress on soil microorganisms, thus changing the microbial community structure (Waldrop & Zak, 2006). Soil moisture changes drive microbial activity by regulating the oxygen and water content in the soil, and ultimately affect nutrient utilization and soil function (Jiao, Lu & Wei, 2022b). The results of RDA analysis in this study showed that pH and SWC were the key factors determining the microbial community structure (Figs. 2G–2H). In general, soil properties can significantly regulate soil multifunctionality by affecting nutrient availability and microbial communities.

### Effects of soil microbial diversity and network complexity on soil multifunctionality

Our results showed that, in addition to soil properties, soil microbial community structure also plays a crucial role in regulating SMF. Although microbial diversity and network complexity were positively correlated with SMF, the relationship between microbial diversity and SMF was more significant (Fig. 4, Figs. S8 and S9). We found that microbial diversity explained SMF more than network complexity (Fig. 2). This may be because higher biodiversity can provide more protection for the maintenance of multiple functions under various temporal and spatial conditions (Wagg et al., 2014). In addition, soil microbial diversity is closely related to network complexity. The former is the basis for the formation of microbial networks and is crucial to the function of microbial networks (Chen et al., 2022a). Delgado-Baquerizo et al. (2020) also found a significant positive effect of soil microbial diversity on SMF in the study of farmland, grassland and forest ecosystems. However, some studies have found that there is a significant negative correlation between microbial diversity and SMF (Delgado-Baquerizo et al., 2020). The possible reason is that the effects of different environmental factors on soil microbial diversity and SMF will offset or cooperate with each other, thus concealing the true relationship between soil microbial diversity and ecosystem function (Delgado-Baquerizo et al., 2020). For example, environmental factors that have both positive or negative effects on microbial diversity and ecological functions will lead to positive correlations, while environmental factors that have opposite effects on both will lead to negative correlation (Hillebrand & Matthiessen, 2009).

Notably, the relative importance of bacterial and fungal diversity to SMF is not consistent. There was a significant positive correlation between bacterial diversity and SMF (*P* < 0.05), while the relationship between fungi was not significant (Fig. 4, Fig. S8), which indicated that bacterial diversity could better predict and positively affect SMF than network complexity. The abundance and diversity of bacteria are much higher than that of fungi, which is one of the main reasons why the relationship between bacterial diversity and SMF is stronger than that of fungi (Jia et al., 2024). Secondly, the bacterial community has higher activity in the soil and grows relatively fast, making them more resistant to interference and disturbance (Zhang et al., 2021). Moreover, this pattern likely reflects fundamental functional differences between bacteria and fungi. Bacteria possess a broader metabolic repertoire for key functions underpinning SMF, such as nitrification (performed by ammonia-oxidizing bacteria), labile carbon decomposition, and phosphorus solubilization (Delgado-Baquerizo et al., 2016; Philippot, Griffiths Bryan & Langenheder, 2021). In contrast, fungi are more specialized in recalcitrant organic matter degradation (such as lignin) but contribute less directly to nutrient mineralization rates that dominate our SMF index (Huang et al., 2023).

It should be noted that, due to limited sample size, this study constructed microbial co-occurrence networks using composite samples pooled across soil depth layers. This approach may mask depth-specific microbial interactions and reduce the spatial resolution of the network. In addition, sampling at a single time point cannot fully capture the seasonal dynamics of microbial communities and soil functions, and amplicon sequencing may be subject to biases such as primer preference or PCR amplification artifacts. Therefore, future studies could further validate and extend our findings by increasing replication, conducting multi-season sampling, and integrating metagenomic approaches.

## Conclusions

Soil multifunctionality in the subalpine grasslands declined significantly along the soil depth. Both abiotic (soil properties) and biotic (microbial diversity) factors jointly governed soil multifunctionality. Specifically, soil properties and microbial diversity were the dominant factors affecting SMF. Bacterial diversity showed significant positive correlations with each SMF, whereas fungal diversity exhibited no significant relationship.

##  Supplemental Information

10.7717/peerj.20734/supp-1 Supplemental Information 1 Supplemental figures and tables

10.7717/peerj.20734/supp-2 Supplemental Information 2 Raw data

## Additional Information and Declarations

### Data Availability

The following information was supplied regarding data availability: The sequences are available at NCBI: PRJNA932581.
