# Supplementary material for: The soil depth determines soil multifunctionality via shaping the soil properties and microbial diversity
Source: PeerJ. 2026 Feb 16;14:e20734. doi: 10.7717/peerj.20734 (PMC12919322; doi:10.7717/peerj.20734)
Supplement: Supplemental Information 1 [file peerj-14-20734-s001.docx]

**The soil depth determines soil multifunctionality via shaping the soil properties and microbial diversity**

Xiaoxia Liang ^1^, Yun xiao Zhao ^2^, Yanhua Song ^2^, Baofeng Chai ^1*^, Tong Jia ^1*^

^1^ Key Laboratory of Shanxi Province for Ecological Restoration of Loess Plateau, Institute of Loess Plateau, Shanxi University, Taiyuan, China

^2^ School of History and Culture, Shanxi University, Taiyuan, China

**Supplementary Method**

**PCR amplifications**

PCRs were repeated for each sample, and the products were mixed using a PCR instrument ABI GeneAmp ® 9700 (ABI, U.S.) and detected by 2 % agarose gel electrophoresis. These PCR products were then purified with an AxyPrep DNA Gel Extraction Kit (Axygen Biosciences, Union City, CA, USA) and quantified using a Quantus™ Fluorometer (Promega, USA). Finally, the amplified products of soil bacterial and fungal were sequenced using the MiSeqPE 300 platform of Shanghai Meiji Biomedical Technology Co., Ltd.for high-throughput gene sequencing.

The original sequencing data were quality-controlled and spliced by fastp (Chen *et al.*, 2018) and FLASH(Magoč *et al.*, 2011b) software to eliminate chimeras. According to the operation instructions, UPARSE software was used to cluster the operational taxonomic units (OTUs) of high-quality sequences at a threshold of 97 % (Edgar, 2013 ; Stackebrandt *et al.*, 1994). RDP classifier (Wang *et al.*, 2007) was used to annotate the OTU sequence. The bacterial sequence was aligned with the SILVA 138 database, and the fungal sequence was aligned with the UNITE8.0 database. The alignment threshold was 70 %. In order to avoid errors caused by different sequencing depths, the minimum number of sample sequences is used for leveling.

**High-throughput sequencing data analysis**

Raw FASTQ files were de-multiplexed using an in-house perl script, and then quality-filtered by fastp version 0.19.6(Chen *et al.*, 2018) and merged by FLASH version 1.2.7(Magoč *et al.*, 2011a) with the following criteria: (i) the reads were truncated at any site receiving an average quality score of <20 over a 10 bp sliding window, and the truncated reads shorter than 50 bp were discarded, reads containing ambiguous characters were also discarded; (ii) only overlapping sequences longer than 10 bp were assembled according to their overlapped sequence. The maximum mismatch ratio of overlap region is 0.2. Reads that could not be assembled were discarded; (iii) Samples were distinguished according to the barcode and primers, and the sequence direction was adjusted, exact barcode matching, 2 nucleotide mismatch in primer matching. Then the optimized sequences were clustered into operational taxonomic units (OTUs) using UPARSE 11.0.667 (Edgar, 2013) with 97% sequence similarity level. The most abundant sequence for each OTU was selected as a representative sequence. The OTU table was manually filtered, i.e., chloroplast sequences in all samples were removed. To minimize the effects of sequencing depth on alpha and beta diversity measure, the number of 16S rRNA gene sequences from each sample were rarefied to 20,000, which still yielded an average Good’s coverage of 99.09%, respectively.

For high-throughput sequencing, DNA was extracted from each of the three independent plots (biological replicates, n = 3 per soil layer). Each DNA extract was subjected to a single PCR amplification and sequencing run (no technical replicates), following standard protocols for amplicon sequencing.

**Microbial co-occurrence network construction and analyses**

The text describes various network metrics, including nodes, edges, average degree, network density, modularity, average clustering coefficient, and average path length, positive correlation, negative correlation and so on. Among them, the greater the value of average degree and network density, the closer the network connectivity of the indication network, while the smaller the value of average path length and network diameter, the higher the connection degree of the indication network; the higher the modular value, the stronger the ability of the microbial community to resist external interference; the larger the average clustering coefficient, the stronger the orderliness and connectivity of the microbial community (Evans *et al.*, 2022). The value of betweeness centrality can characterize the importance of network nodes to the tight connectivity of the network and the availability of resources. The greater the value of betweeness centrality, the higher the connectivity of the node in the network.

**Assessing soil multifunctionality**

To minimize the influence of non-random selection of soil functions, we used both the average value (Eq. 1) and multiple thresholds methods (Eq. 2) to quantify soil multifunctionality via the “multifunc” R package (v0.8.0) (Byrnes *et al.*, 2014).

$Soil multifunctionality=\frac{1}{F}\sum_{i=1}^{F} g(r(f_{i}))$ (1)

$f_{i}$is the measurement of a single soil function $i$; $r$is a mathematical function that transforms $f_{i}$ to be positive; $g$ is the mathematical function to transform soil function i to [0,1] by the min-max normalization method(Byrnes *et al.*, 2014); and $F$ is the total number of soil functions that were measured.

While the average value method offers a straightforward and interpretable metric for assessing soil multifunctionality, it fails to account for potential trade-offs among these functions and assumes interchangeability of functionalities. To overcome these limitations, we also quantified multifunctionality using the multiple thresholds method. The multiple thresholds method captures the number of functions that simultaneously exceed different thresholds of the maximum observed value of each function and evaluates whether more (or fewer) functions are performing simultaneously at high (or low) levels, providing a more nuanced and comprehensive understanding of soil multifunctionality(Byrnes *et al.*, 2014).

$Soil multifunctionality=\sum_{i=1}^{F} (r_{i}{(f)}_{i}>t_{i})$ (2)

In other words, soil multifunctionality using the multiple thresholds method was the number of soil functions with standardized values greater than a given threshold ($t_{i}$). This approach involved testing the effect of microbial diversity on soil multifunctionality across the full range of soil multifunctionality thresholds between 5% and 99%, providing the following indices: Tmin represents the minimum threshold—the minimum functional threshold where the multifunctionality begins to be affected by diversity—when the slope of the relationship between function and diversity was significantly different from 0; Tmax represents the maximum threshold—the multifunctionality beyond Tmax is not affected by diversity and there is no difference between the slope of the linear regression relationship and 0; Tmde represents the threshold of maximum diversity effect—the multifunctionality of the threshold proportion is most affected by diversity and the slope reaches the maximum; and Rmde represents the realised maximum effect of diversity—the relationship strength between multifunctionality and diversity (i.e. slope) at Tmde.

**Data analysis**

To address multicollinearity among environmental predictors, we calculated variance inflation factors (VIF) for all variables using the “vif()” function in the R package car. Variables were iteratively removed, starting with the one exhibiting the highest VIF, until all remaining variables had VIF < 5, which was a commonly used threshold in ecological studies to ensure model stability(Zuur *et al.*, 2010). The selection principle of RDA or CCA model: DCA (detrended correspondence analysis) analysis was performed using species-sample data (97 % similarity sample OTU table) to see the size of the first axis of the Lengths of gradient in the analysis results. If it is greater than 4.0, CCA should be selected; if between 3.0-4.0, RDA and CCA can be selected; if less than 3.0, the results of RDA are better than CCA.

**Supplementary table and figure captions**

**Fig. S1** The physicochemical properties of different soil depths in the subalpine grassland of Heyeping in Luya Mountain. The error bar in the figure represents the standard deviation, and different letters indicate significant differences between different soil depths.

**Fig. S2** Upset analysis of OTU level of microbial community in different soil depths. (a) Bacterial community; (b) Fungal community. The left area in the figure shows the number of OTUs for each soil layer. The black line below represents different soil layers and their collections. The column chart above shows the corresponding number of OTUs for each collection. The circular collection on the right shows a visualization of the number of OTUs for each soil layer.

**Fig.S3** Top 12 soil (a) bacterial and (b) fungal community composition at class level in different soil depths of subalpine grassland in Luya Mountain.

**Fig. S4** Differences analysis in the composition of the (a-b) soil bacterial and (c-d) fungal communities of the top 12 at the phylum level and the top 15 at the class level along the soil depths.

**Fig. S5** Topological properties of microbial co-occurrence network in different soil layers.

**Fig. S6** Mental analysis between bacteria (a-b) and fungi (c-d) top 10 key taxa and diversity with soil physicochemical properties and enzyme activities.

**Fig. S7** Structural models of community structure and diversity drivers for (ac) bacteria and (bd) fungi in subalpine meadow soils. The upper panel ab shows the structural equation model, with red lines representing positive effects, blue lines representing negative influence. The thickness of the line represents the standardized path coefficient, and the value above it is the standardized path coefficient. The R^2^ value represents the variance ratio explained by each driving factor. The lower panel cd is the standardized total effect. The same below.

**Fig. S8** Relationships of soil bacterial and fungal network complexity with the three soil multifunctionality.

**Fig. S9** Relationship between fungal diversity and function with different thresholds in different ecosystem multifunctionality categories.

**Fig. S10** Correlation and explanation of soil physicochemical properties, enzyme activity and biotic factor with the three types of soil multifunctionality based on random forest model.

**Table S1** Univariate ANOVA of soil enzyme activities at different depths in the subalpine Carex grassland of Luya Mountain

**Table S2** Topological properties of microbial community co-occurrence network at different soil layers

**Table S3** Key microbial OTUs in co-occurrence network at different soil layers

**Table S4** Mental test analysis of environmental factors and microbial communities diversity


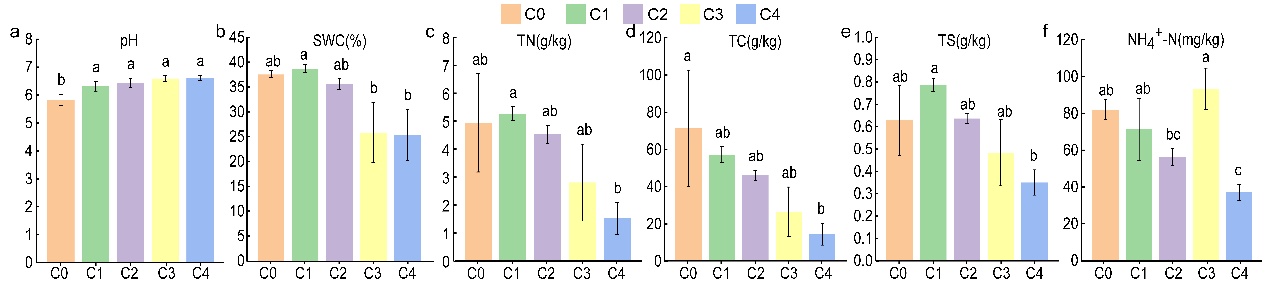


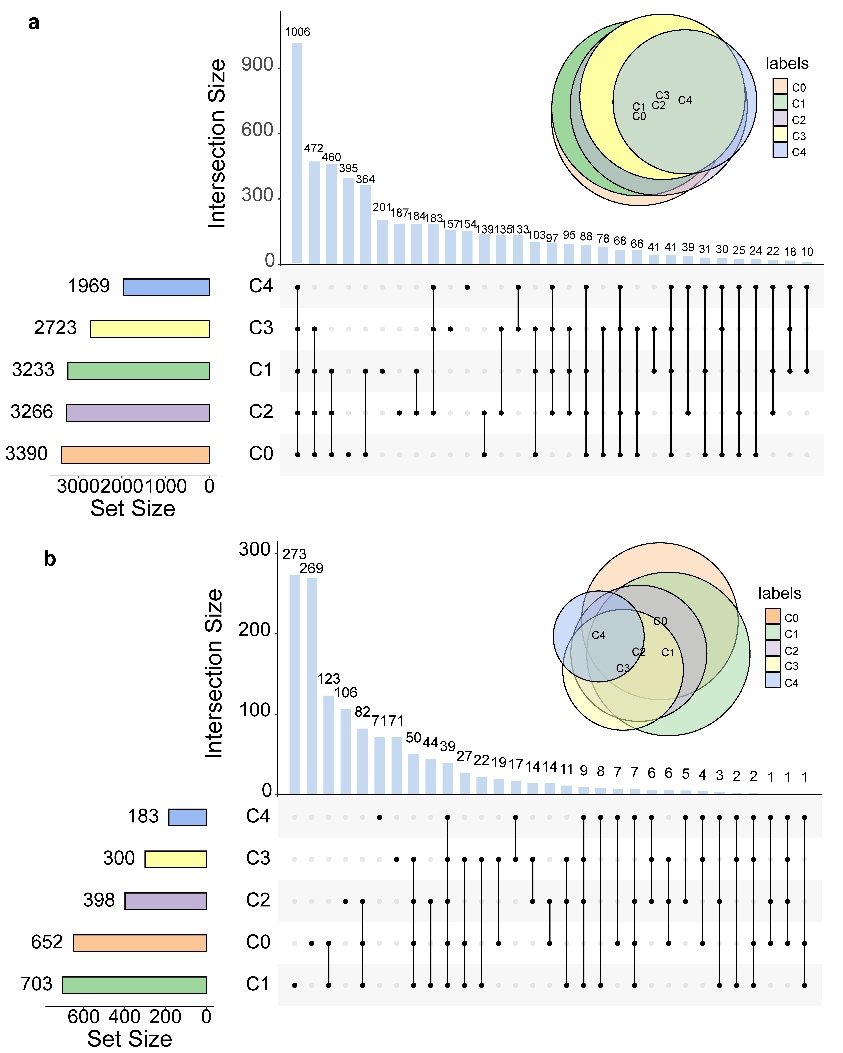
**Fig. S1** The physicochemical properties of different soil depths in the subalpine grassland of Heyeping in Luya Mountain. The error bar in the figure represents the standard deviation, and different letters indicate significant differences between different soil depths.

**Fig. S2** Upset analysis of OTU level of microbial community in different soil depths. (a) Bacterial community; (b) Fungal community. The left area in the figure shows the number of OTUs for each soil layer. The black line below represents different soil layers and their collections. The column chart above shows the corresponding number of OTUs for each collection. The circular collection on the right shows a visualization of the number of OTUs for each soil layer.


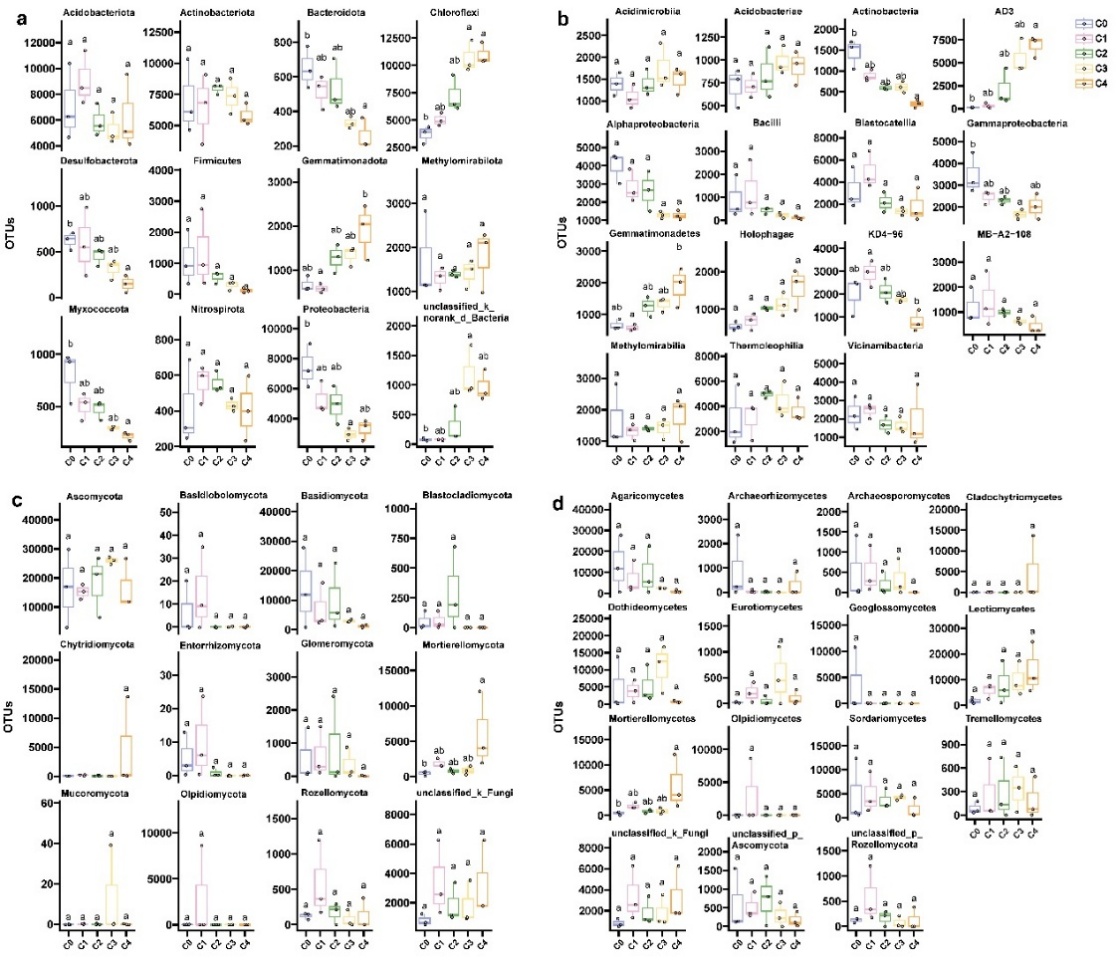

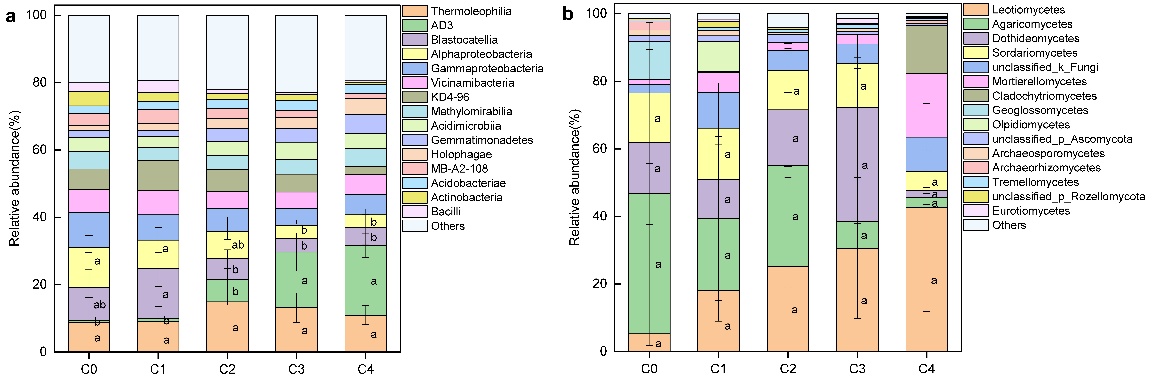
**Fig.S3** Top 12 soil (a) bacterial and (b) fungal community composition at class level in different soil depths of subalpine grassland in Luya Mountain.

**Fig. S4** Differences analysis in the composition of the (a-b) soil bacterial and (c-d) fungal communities of the top 12 at the phylum level and the top 15 at the class level along the soil depths.


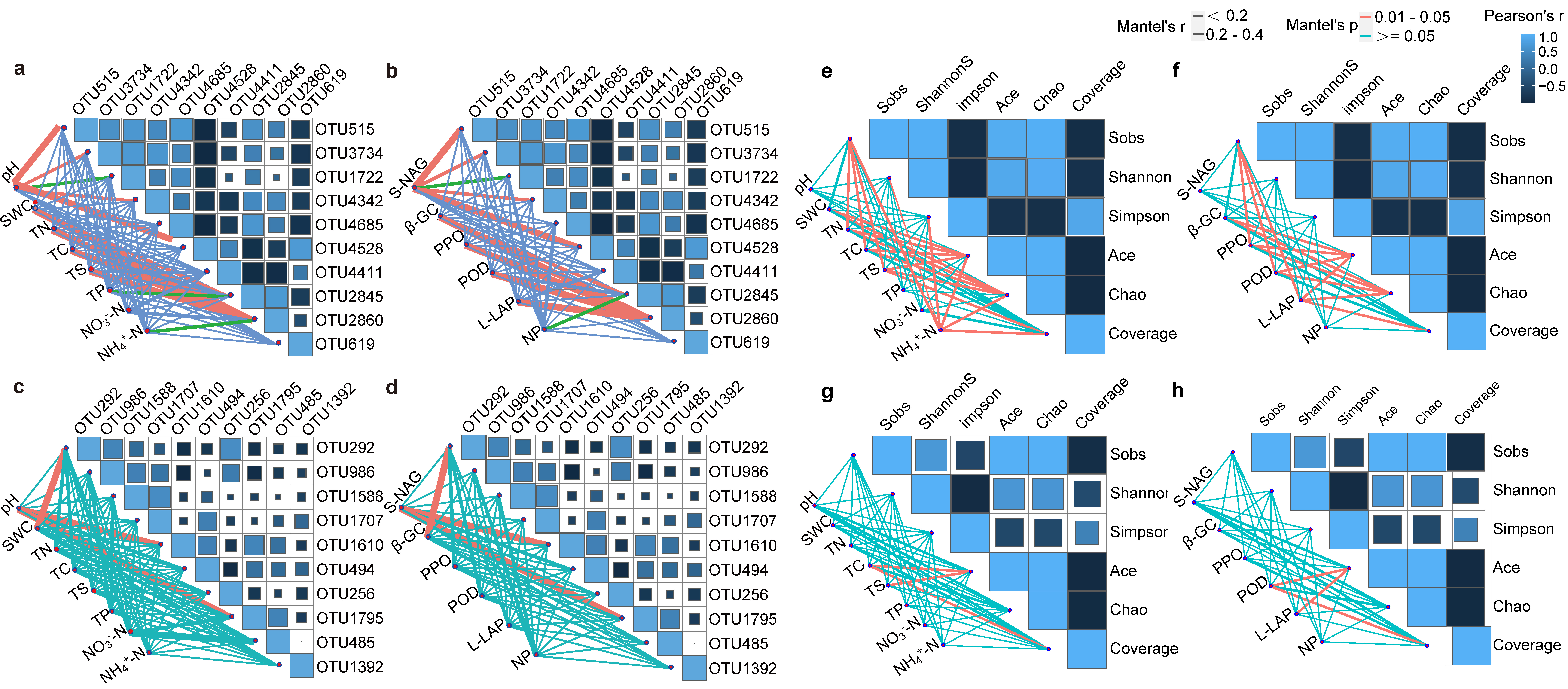

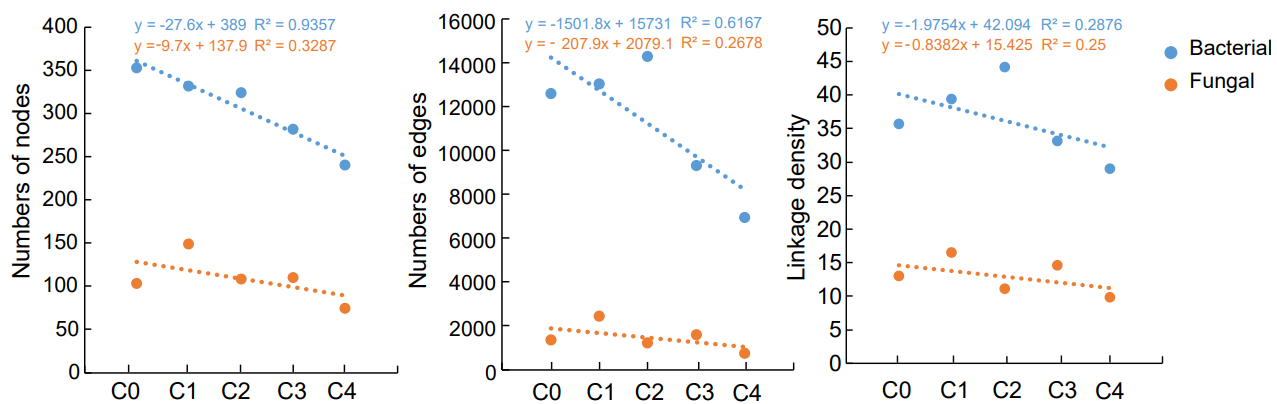
**Fig. S5** Topological properties of microbial co-occurrence network in different soil layers.

**

Fig. S6** Mental analysis between bacterial (a-b) and fungal (c-d) top 10 key taxa and diversity with soil physicochemical properties and enzyme activities.

**Fig.** **S7** Structural models of community structure and diversity drivers for (ac) bacteria and (bd) fungi in subalpine meadow soils. The upper panel ab shows the structural equation model, with red lines representing positive effects, blue lines representing negative influence. The thickness of the line represents the standardized path coefficient, and the value above it is the standardized path coefficient. The R^2^ value represents the variance ratio explained by each driving factor. The lower panel
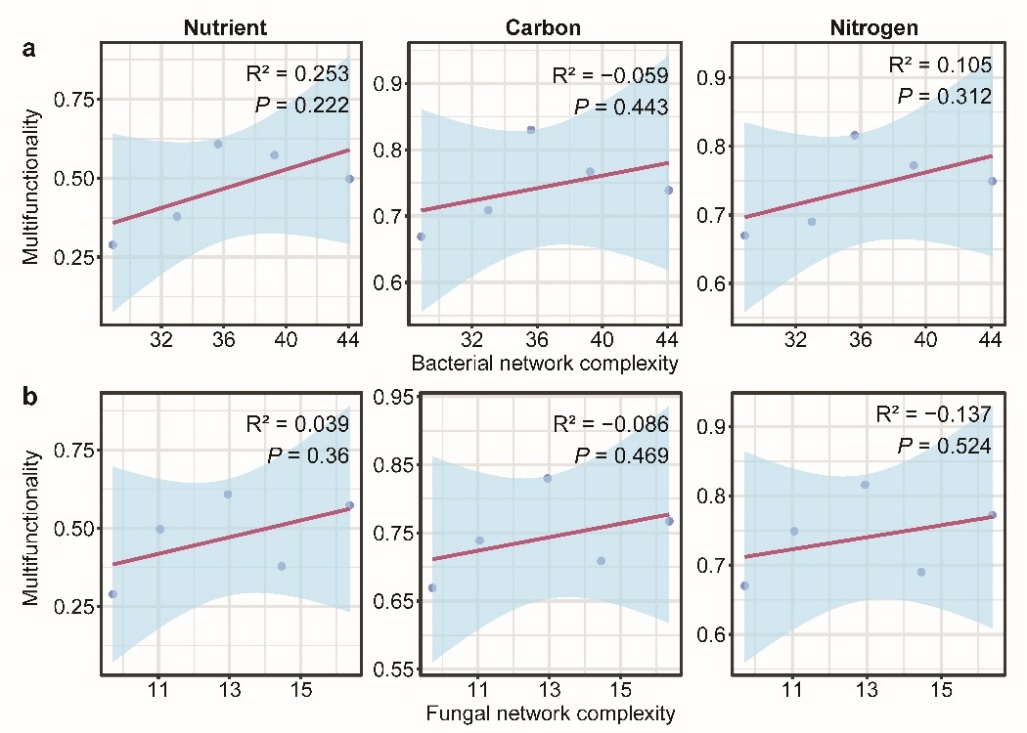
cd is the standardized total effect. The same below.


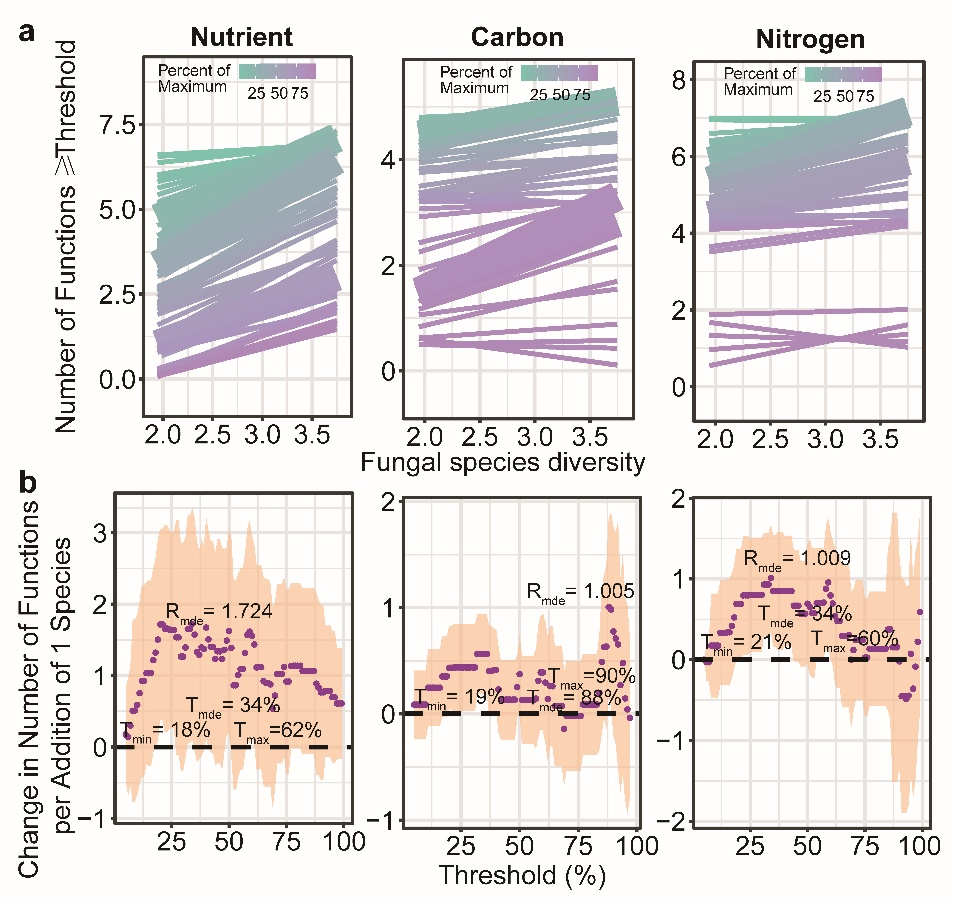
**Fig. S8** Relationships of soil bacterial and fungal network complexity with the three soil multifunctionality.

**Fig. S9** Relationship between fungal diversity and function with different thresholds in different ecosystem multifunctionality categories. (a) The relationship between fungal diversity and the number of functions is equal to or greater than the observed functions of different proportions. The solid line is the fitting relationship of the GLMs; the gradient colour represents the maximum proportion of functions. (b) The slope between fungal diversity and different threshold functions. The purple solid point is the fitting value of the GLMs; the shaded part represents the ±1 confidence interval. T_min_ represents the minimum threshold—the minimum functional threshold where the multifunctionality begins to be affected by diversity— when the slope of the relationship between function and diversity was significantly different from 0; T_max_ represents the maximum threshold—the multifunctionality beyond T_max_ is not affected by diversity and there is no difference between the slope of the linear regression relationship and 0; T_mde_ represents the threshold of maximum diversity effect—the multifunctionality of the threshold proportion is most affected by diversity and the slope reaches the maximum; and R_mde_ represents the realised maximum effect of diversity—the relationship strength between multifunctionality and
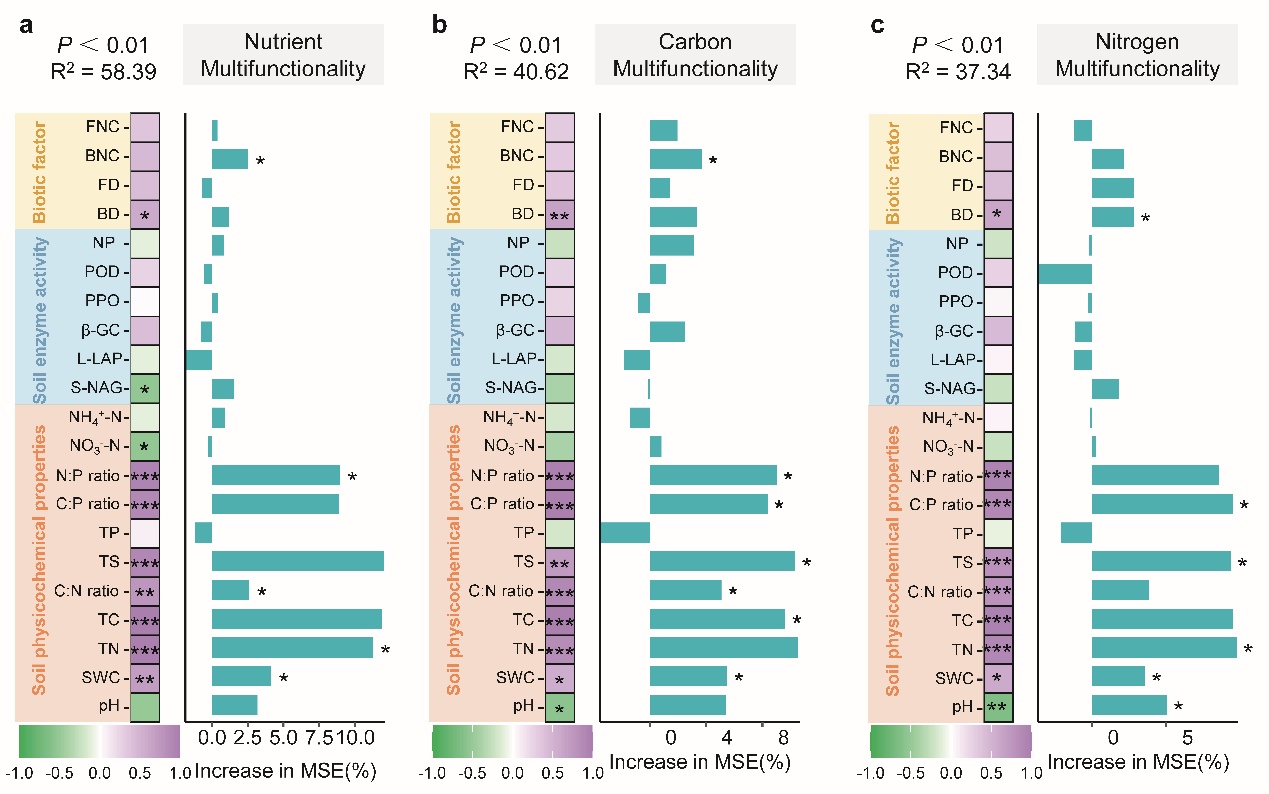
diversity (i.e. slope) at T_mde_.

**Fig. S10** Correlation and explanation of soil physicochemical properties, enzyme activity and biotic factor with the three types of soil multifunctionality based on random forest model. (Significant levels are shown at: * *P* < 0.05, * * *P* < 0.01, * * * *P* < 0.001)

**Table S1** Univariate ANOVA of soil enzyme activities at different depths in the subalpine *Carex* grassland of Luya Mountain.

| Soil depths | S-NAG (IU/g) | β-GC (IU/g) | PPO (IU/g) | POD (mU/g) | L-LAP (IU/g) | NP (IU/g) |
| --- | --- | --- | --- | --- | --- | --- |
| C0 | 118.839±5.914 | 36.847±3.525 | 235.626±7.478 | 14.187±0.707 | 133.917±7.674 | 36.611±1.148 |
| C1 | 114.256±5.527 | 38.608±1.168 | 224.235±12.733 | 13.709±0.418 | 133.385±7.567 | 40.004±2.887 |
| C2 | 114.346±9.581 | 36.93±1.81 | 228.197±10.016 | 13.844±0.465 | 140.412±11.303 | 38.713±2.883 |
| C3 | 116.952±9.766 | 38.14±0.719 | 238.267±17.728 | 13.782±0.489 | 142.328±5.734 | 37.001±1.056 |
| C4 | 123.152±3.928 | 35.885±2.241 | 228.692±20.8 | 13.512±0.639 | 139.56±8.516 | 39.914±0.869 |

**Table S2** Topological properties of microbial community co-occurrence network at different soil layers.

| Topological properties | Bacterial community | | | Fungal community | | |
| --- | --- | --- | --- | --- | --- | --- |
|  | C0+C1+C2+C3+C4 | C1+C2 | C3+C4 | C0+C1+C2+C3+C4 | C1+C2 | C3+C4 |
| Nodes | 337 | 341 | 268 | 158 | 147 | 120 |
| Edges | 10069 | 7111 | 2986 | 896 | 1524 | 1029 |
| Average Degree | 59.757 | 41.707 | 22.284 | 11.342 | 20.735 | 17.150 |
| Network diameter | 5 | 7 | 6 | 11 | 12 | 10 |
| Network Density | 0.178 | 0.123 | 0.083 | 0.072 | 0.142 | 0.144 |
| Modularity | 0.3 | 0.452 | 0.587 | 0.847 | 0.776 | 0.706 |
| Average Clustering Coefficient | 0.582 | 0.579 | 0.563 | 0.849 | 0.914 | 0.935 |
| Average Path Length | 2.159 | 2.574 | 2.820 | 4.929 | 4.885 | 4.225 |
| Positive correlation | 62.05% | 66.49% | 72.30% | 100% | 99.74% | 99.42% |
| Negative correlation | 37.95% | 33.51% | 27.70% | 0 | 0.26% | 0.58% |

**Table S3** Key microbial OTUs in co-occurrence network at different soil layers.

|  | Soil depth | OTU | Phylum | Class | Betweeness centrality |
| --- | --- | --- | --- | --- | --- |
| Bacterial community | C0+C1+C2+C3+C4 | OTU515 | Actinobacteriota | Thermoleophilia | 1212.518 |
|  |  | OTU3734 | Actinobacteriota | Thermoleophilia | 808.538 |
|  |  | OTU1722 | Actinobacteriota | Thermoleophilia | 749.504 |
|  |  | OTU4342 | Proteobacteria | Gammaproteobacteria | 731.626 |
|  |  | OTU4685 | Chloroflexi | KD4-96 | 720.637 |
|  | C1+C2 | OTU2091 | Actinobacteriota | Acidimicrobiia | 1185.008 |
|  |  | OTU3967 | Proteobacteria | Gammaproteobacteria | 1127.600 |
|  |  | OTU876 | Chloroflexi | Chloroflexia | 1035.239 |
|  |  | OTU1128 | Actinobacteriota | Acidimicrobiia | 1028.721 |
|  |  | OTU3780 | Actinobacteriota | Thermoleophilia | 967.459 |
|  | C3+C4 | OTU4548 | Methylomirabilota | Methylomirabilia | 1066.450 |
|  |  | OTU188 | Chloroflexi | JG30-KF-CM66 | 1014.735 |
|  |  | OTU1841 | Proteobacteria | Gammaproteobacteria | 936.011 |
|  |  | OTU4685 | Chloroflexi | KD4-96 | 919.817 |
|  |  | OTU2589 | Actinobacteriota | Thermoleophilia | 842.605 |
| Fungal community | C0+C1+C2+C3+C4 | OTU292 | Ascomycota | Sordariomycetes | 2423.112 |
|  |  | OTU986 | Basidiomycota | Tremellomycetes | 2151.518 |
|  |  | OTU1588 | unclassified_k_Fungi | unclassified_k_Fungi | 2054.058 |
|  |  | OTU1707 | Ascomycota | Leotiomycetes | 1957.082 |
|  |  | OTU1610 | Basidiomycota | Agaricomycetes | 1732.009 |
|  | C1+C2 | OTU494 | Ascomycota | Sordariomycetes | 3300.000 |
|  |  | OTU908 | Rozellomycota | unclassified_p_Rozellomycota | 3294.000 |
|  |  | OTU484 | Basidiomycota | Agaricomycetes | 3286.298 |
|  |  | OTU935 | Mortierellomycota | Mortierellomycetes | 2376.000 |
|  |  | OTU1631 | Ascomycota | Leotiomycetes | 2314.000 |
|  | C3+C4 | OTU597 | Ascomycota | Dothideomycetes | 1443.000 |
|  |  | OTU1785 | Mortierellomycota | Mortierellomycetes | 1315.000 |
|  |  | OTU1554 | Ascomycota | Sordariomycetes | 1219.000 |
|  |  | OTU1396 | Ascomycota | Sordariomycetes | 1155.000 |
|  |  | OTU1507 | Ascomycota | Leotiomycetes | 1083.000 |

**Table S4** Mental test analysis of environmental factors and microbial community diversity.

| Factor | Bacterial community | | Fungal community | |
| --- | --- | --- | --- | --- |
|  | r | *P* | r | *P* |
| pH | 0.3627 | **0.004** | 0.2801 | **0.004** |
| SWC | 0.2148 | **0.044** | 0.1653 | 0.12 |
| TN | 0.2704 | **0.014** | 0.3267 | **0.003** |
| TC | 0.3258 | **0.003** | 0.3359 | **0.001** |
| TS | 0.2406 | **0.021** | 0.2679 | **0.006** |
| TP | 0.0589 | 0.283 | -0.0551 | 0.631 |
| NO_3_^-^-N | -0.0137 | 0.505 | -0.0784 | 0.714 |
| NH_4_^+^-N | 0.0517 | 0.232 | 0.0743 | 0.266 |
| S-NAG | -0.0869 | 0.832 | -0.1195 | 0.894 |
| β-GC | 0.0626 | 0.22 | 0.1314 | 0.115 |
| PPO | -0.0311 | 0.612 | 0.0194 | 0.445 |
| POD | 0.0539 | 0.301 | 0.1205 | 0.16 |
| L-LAP | -0.1039 | 0.892 | -0.1076 | 0.857 |
| NP | -0.0045 | 0.445 | 0.0748 | 0.275 |

References

**Byrnes J.E.K., Gamfeldt L., Isbell F., et al. 2014.** Investigating the relationship between biodiversity and ecosystem multifunctionality: challenges and solutions. *Methods in Ecology and Evolution* **5**: 111-124 <https://doi.org/https://doi.org/10.1111/2041-210X.12143>

**Chen S., Zhou Y., Chen Y., et al. 2018.** fastp: an ultra-fast all-in-one FASTQ preprocessor. *Bioinformatics* **34**: i884-i890 <https://doi.org/10.1093/bioinformatics/bty560>

**Edgar R.C. 2013.** UPARSE: highly accurate OTU sequences from microbial amplicon reads. *Nature Methods* **10**: 996-998 <https://doi.org/10.1038/nmeth.2604>

**Evans T.S., and Chen B. 2022.** Linking the network centrality measures closeness and degree. *Communications Physics* **5**: 172 <https://doi.org/10.1038/s42005-022-00949-5>

**Magoč T., and Salzberg S. 2011a.** FLASH: fast length adjustment of short reads to improve genome assemblies. *Bioinformatics* **27**: 2957-2963 <https://doi.org/10.1093/bioinformatics/btr507>

**Magoč T., and Salzberg S.L. 2011b.** FLASH: fast length adjustment of short reads to improve genome assemblies. *Bioinformatics* **27**: 2957-2963 <https://doi.org/10.1093/bioinformatics/btr507>

**Stackebrandt E., and Goebel B.M. 1994.** A PLACE FOR DNA-DNA REASSOCIATION AND 16S RIBOSOMAL-RNA SEQUENCE-ANALYSIS IN THE PRESENT SPECIES DEFINITION IN BACTERIOLOGY. *International Journal of Systematic Bacteriology* **44**: 846-849 <https://doi.org/10.1099/00207713-44-4-846>

**Wang Q., Garrity G.M., Tiedje J.M., et al. 2007.** Naive Bayesian classifier for rapid assignment of rRNA sequences into the new bacterial taxonomy. *Appl Environ Microbiol* **73**: 5261-5267 <https://doi.org/10.1128/aem.00062-07>

**Zuur A.F., Ieno E.N., and Elphick C.S. 2010.** A protocol for data exploration to avoid common statistical problems. *Methods in Ecology and Evolution* **1**: 3-14 <https://doi.org/https://doi.org/10.1111/j.2041-210X.2009.00001.x>
